# On the Best Way to Cluster NCI-60 Molecules

**DOI:** 10.3390/biom13030498

**Published:** 2023-03-08

**Authors:** Saiveth Hernández-Hernández, Pedro J. Ballester

**Affiliations:** 1 Cancer Research Center of Marseille (INSERM U1068, Institut Paoli-Calmettes, Aix-Marseille Université UM105, CNRS UMR7258), 13009 Marseille, France; 2 Department of Bioengineering, Imperial College London, London SW7 2AZ, UK

**Keywords:** NCI-60 panel, small molecules, clustering, model validation

## Abstract

Machine learning-based models have been widely used in the early drug-design pipeline. To validate these models, cross-validation strategies have been employed, including those using clustering of molecules in terms of their chemical structures. However, the poor clustering of compounds will compromise such validation, especially on test molecules dissimilar to those in the training set. This study aims at finding the best way to cluster the molecules screened by the National Cancer Institute (NCI)-60 project by comparing hierarchical, Taylor–Butina, and uniform manifold approximation and projection (UMAP) clustering methods. The best-performing algorithm can then be used to generate clusters for model validation strategies. This study also aims at measuring the impact of removing outlier molecules prior to the clustering step. Clustering results are evaluated using three well-known clustering quality metrics. In addition, we compute an average similarity matrix to assess the quality of each cluster. The results show variation in clustering quality from method to method. The clusters obtained by the hierarchical and Taylor–Butina methods are more computationally expensive to use in cross-validation strategies, and both cluster the molecules poorly. In contrast, the UMAP method provides the best quality, and therefore we recommend it to analyze this highly valuable dataset.

## 1. Introduction

In the past decades, artificial intelligence (AI) has been used to develop predictive models with a wide range of applications in biomedicine and healthcare [1]. In particular, machine learning (ML)—a subarea of AI—has become an important component in early drug discovery, for instance, by developing quantitative structure–activity relationship (QSAR) models [2]. The use of ML-based models in drug discovery has been possible due to the availability of preclinical data that can be reused to build and validate predictive models. Such is the case of the National Cancer Institute (NCI-60) human tumor cell lines screen, which since 1990 has been used by the cancer research community to find compounds with potential anticancer activity [3].

Although ML-based models have been extensively used, several challenges remain to be overcome along the drug-design pipeline, one of them related to model performance on unseen compounds. Indeed, many articles fail to account for the nearly inevitable reduction in predictive ability that may occur when something that is a useful predictor in one data set is not as useful in another dataset [4]. Models that perform well on an independent data set can be achieved using model validation strategies such as bootstrap or k-fold cross-validation (CV) [4]. More demanding model validation strategies include asymmetric validation embedding (AVE) [5], leave-dissimilar-target-out (LDTO) CV [6], leave-one-cell-line-out (LOCO) CV, leave-one-tissue-out (LOTO) CV, leave-one-compound-cluster-out (LOCCO) CV [7], and those using other similarity metrics between training and test data instances [8]. These complex model-validation methods pose additional challenges [9], particularly the LOCCO CV approach, which inherits the challenges of any clustering method.

Clustering methods are widely explored unsupervised ML-based algorithms whose aim is to discover the underlying structure or patterns existing in a given dataset. The clustering of biological entities, such as small molecules, can be performed using different approaches, including hierarchical clustering (HC), distribution-based clustering, and density-based clustering [10]. For example, several clustering algorithms, including hierarchical, Taylor–Butina, and UMAP clustering, have been compared on 29 data sets with between 100 and 5000 small molecules [11]. In addition, hierarchical clustering has been used to cluster molecules from the PubChem database [12], and Taylor–Butina clustering has been used to cluster molecules from the MolPort database [13].

A clustering analysis in a virtual chemical database can be used to quantify the diversity of compounds, which is relevant in several areas of chemistry such as in high-throughput screening (HTS) [14] and in QSAR-based virtual screening predictions [13]. In addition, the resulting clustering of compounds could lead to a comprehensive understanding of the underlying mechanism of action (MOA) of the drugs [15]. However, clustering chemical compounds also poses a challenge in terms of their representation. This is because chemical compounds can be represented in different ways, which can result in different clustering outcomes. It is possible for two compounds with different molecular structures to have comparable molecular descriptors or fingerprints, leading to them being clustered together using those representations. Similarly, two compounds with similar molecular structures may have different molecular descriptors or fingerprints, resulting in them being clustered separately using those representations. Structural fingerprints, such as the 1024-bit Morgan fingerprint, offer several advantages over other molecular representations for clustering tasks. For instance, they are computationally efficient and can be easily applied to large datasets. Moreover, Morgan fingerprints are robust to small variations in the molecular structure, making them a useful tool in virtual screening, where slight modifications to the molecular structure may occur due to synthetic or computational alterations.

In this study, we aim to evaluate and compare three different methods for clustering the NCI-60 molecules to determine the best-performing algorithm that can be used to generate clusters for model validation, ensuring that the resulting clusters are optimal for use in LOCCO-CV strategies or other model validation strategies. For this, we compare the hierarchical, Taylor–Butina, and UMAP clustering algorithms while tuning their essential hyperparameters. Additionally, we provide a definition of outlier molecules that aligns with the clustering problem we are addressing, allowing us to optimize outlier computation. By applying this definition, we investigate the impact of removing outlier molecules from the NCI-60 panel before clustering. To evaluate the clustering performance, we compute the silhouette coefficient, the Calinski–Harabaz score, and the Davies–Bouldin score for each method. Finally, alongside the clustering metrics, we compute a similarity matrix to achieve a better understanding of the molecules per cluster. The results show that, overall, removing outlier molecules results in better clustering. Moreover, the results show that UMAP clustering outperforms hierarchical and Taylor–Butina clustering.

## 2. Materials and Methods

**Figure 1:**
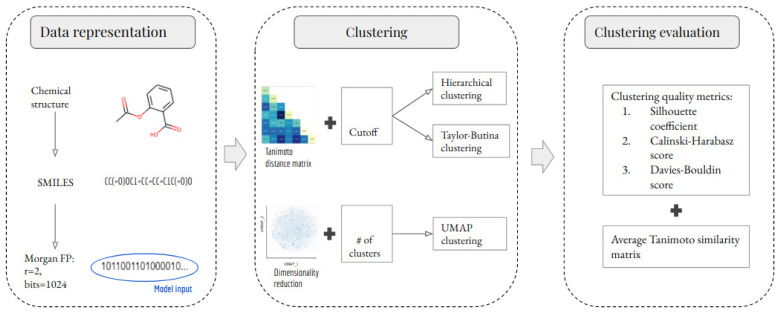
Schematic representation of the development of ML-based models for clustering small molecules. Chemical structures and their SMILES were retrieved from the NCI-60 dataset. SMILES were subsequently preprocessed and used to calculate the MFPs (radius 2 and 1024 bits). The MFPs were used to build and evaluate three clustering methods. Three clustering quality metrics were used to evaluate the clustering performance of each developed model.

The methodology of this study comprises three stages: data representation, clustering, and clustering evaluation. These stages are summarized in Figure 1 and are explained in detail in the following subsections. An important part of this study is to evaluate the impact of removing outlier molecules from the data set. Therefore, this study analyzes two scenarios per clustering method. In the first scenario, all of the unique molecules were clustered. In the second scenario, outlier molecules were removed before clustering. The outlier detection method is explained in Section 3.1.

It is important to note that the outlier detection method and clustering algorithms were applied to the entire set of molecules in the NCI-60 panel and not on a per-cell line basis. Although the clustering output provides information on molecule NSC ID, SMILES representations, and their assigned clusters, it is possible to map the clustering results to each individual cell line.

### 2.1. NCI-60 Dataset

The NCI-60 panel utilizes 60 different human tumor cell lines to identify and characterize novel compounds with growth inhibition or killing of tumor cell lines [3]. These 60 different human tumor cell lines comprise 9 cancer types: leukemia; melanoma; and cancers of the lung, colon, brain, ovary, breast, prostate, and kidney. All of the compounds that are screened have been initially tested in a one-dose assay on the full NCI-60 panel. Compounds showing significant growth inhibition at this assay are then evaluated against the NCI-60 panel in a 5-dose assay [16]. Each compound submitted to the NCI-60 panel for testing and evaluation is identified with a unique registration number called the National Service Center (NSC) ID.

Data quality is crucial in the development of AI/ML models during drug discovery. Therefore, data cleansing is necessary to ensure high-quality data is used to generate these models. To achieve this, we follow the preprocessing stage described by [17]. At the data-representation stage, each molecule is represented by a 1024-bit Morgan fingerprint (MFP) [18] with a radius of 2, which indicates the presence or absence of a particular substructure in the molecule. We chose this fingerprint for several reasons. Firstly, previous research has shown that the choice of fingerprint and metric has little effect on downstream predictions, such as target prediction based on molecular similarity [19]. Furthermore, our choice led to practically the best performance with respect to other fingerprints and metrics, suggesting that it is a near-optimal choice. In [20], the authors showed that the (extended-connectivity) fingerprints of radius 2 and 3 are among the best-performing fingerprints when ranking diverse structures by similarity. Previous research has shown that structural (or rule-based) fingerprints, such as E3FP, Morgan, or topological, should be considered for similarity-based clustering [21]. Finally, the 1024-bit MFP size has been successfully used in retrospective studies [22], for potency prediction [23], similarity searching, and bioactivity classification [24]. Therefore, in this study, the 1024-bit MFPs will be used as features to build the clustering models.

**Figure 2:**
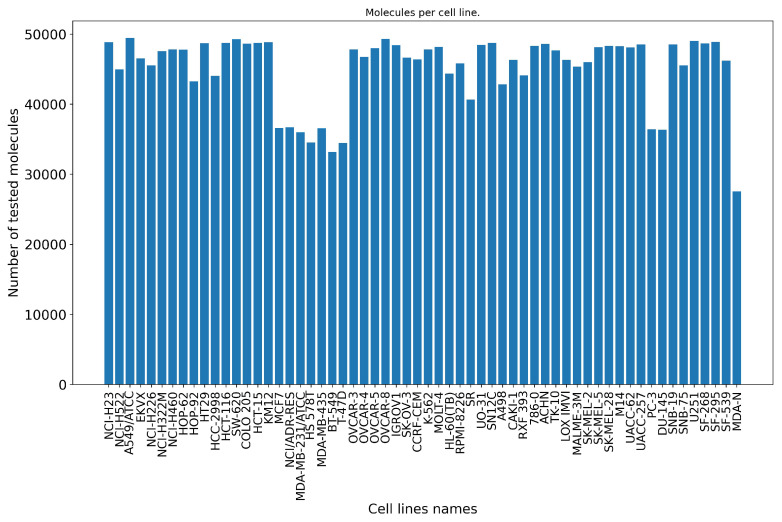
Number of tested molecules per NCI-60 cell line. On average, 45,124 small molecules are screened against each of the 60 cell lines from the NCI-60 panel. Each barplot represents the number of unique molecules (vertical axis) retrieved per cell line (horizontal axis) after the preprocessing stage.

**Figure 3:**
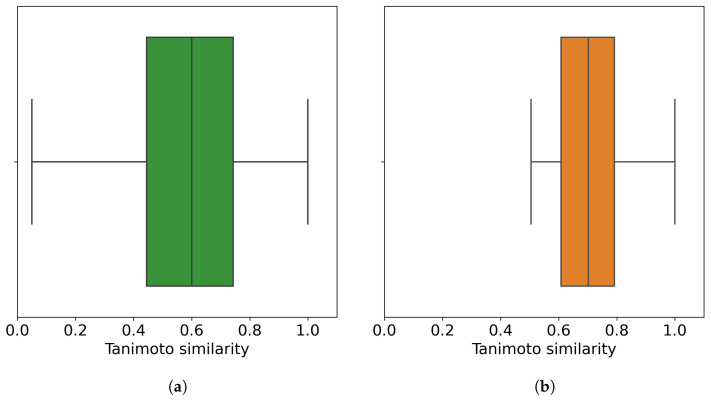
Distribution of the chemical similarity of molecules. (**a**) Before the outlier detection method (all 50,555 molecules); (**b**) after the outlier detection method, where 32,971 non-outlier molecules are retrieved at an outlier cutoff of 0.5. Each point in the corresponding boxplot is a molecule with its similarity to its closest molecule (other than itself). In total, 50% of molecules, either all of the molecules or non-outlier molecules, have their most similar molecule with similarity values less than 0.6 and 0.7, respectively.

After the preprocessing stage, there remain around 2.7 M data points (50,555 small molecules screened against 60 cancer cell lines), which represent a matrix completeness of 89.25%. Figure 2 shows the distribution of the small molecules per cell line. We also analyze the Tanimoto similarity distribution of these small molecules (Figure 3a).

### 2.2. Clustering Methods

Hierarchical clustering consists in building a binary merge tree, starting from the molecules stored at the leaves and merging them until reaching the root of the tree that contains all the molecules of the dataset [25]. The linkage criteria determine the metric used for the merge strategy, for example, single linkage, complete linkage, or Ward linkage. In the context of clustering chemical structure databases, the Ward linkage is commonly used [26], which minimizes the sum of squared differences within all clusters. The graphical representation of the binary merge tree representing the resulting hierarchical clustering is called a dendrogram. The python implementation of this algorithm uses the RDkit library [27], where the first step is to calculate the similarity for each pair of molecules. Then, a distance matrix containing 1−similarity for each pairwise similarity value is created. This distance matrix is the model input (Figure 1). Finally, the linkage criteria used in this study is the Ward linkage.

Taylor–Butina clustering is an algorithm based on exclusion spheres at a given Tanimoto level [28]. The way the clusters are built allows all of the molecules belonging to each cluster to have a Tanimoto value above or equal to the similarity cutoff used. At each iteration, the molecules are visited and labeled, either as a cluster centroid or as a cluster member. A disadvantage of this algorithm is that at the end of the clustering, molecules that have not been labeled are considered as singletons, even if they have neighbors. The reason is that their neighbors have been attracted by a better centroid. With this approach, we benefit from fast clustering since in each iteration only unlabeled molecules are compared, and we avoid the formation of highly heterogeneous clusters [28]. The python implementation of the Taylor–Butina algorithm employs the RDkit [27] library. The distance matrix is calculated in the same way as in hierarchical clustering (Figure 1); then, based on the similarity cutoff given, each molecule is assigned to a cluster id.

The uniform manifold approximation and projection (UMAP) is a non-linear dimensionality reduction algorithm that seeks to learn the manifold structure of the data and find a low-dimensional embedding while preserving the essential topological structure of that manifold [29]. While UMAP has been used for dimensionality reduction [30], it has also been used for clustering [11]. UMAP has four basic parameters to control the impact on the resulting embedding. These are n_neighbors, which controls how UMAP balances local versus global structure in the data; min_dist, which controls how tightly UMAP is allowed to pack points together; n_components, which allows the user to determine the dimensionality of the reduced dimension space we will be embedding the data into; and metric, which controls how distance is computed in the ambient space of the input data. After the dimensionality reduction is completed, this new representation of the molecules is clustered using the AgglomerativeClustering function from the Scikit-learn library [31].

### 2.3. Clustering Evaluation

The last step is the evaluation of the clustering algorithms. In this study, we consider three unsupervised metrics to evaluate the clustering quality results, the silhouette coefficient, the Calinski–Harabasz score, and the Davies–Bouldin score. These metrics are available in the Scikit-learn library [31].

To calculate the silhouette coefficient [32], we first calculate the mean intra-cluster distance $a_{i}$ for each molecule *i* in the cluster $C_{I}$ as follows:

(1) $$a_{i}=\frac{1}{|C_{I}|-1}\sum_{j\in C_{I},i\neq j}d\left(i,j\right),$$

where d(i,j) is the distance between molecules *i* and *j* in the cluster $C_{I}$. Next, we calculate the mean inter-cluster distance $b_{i}$ for each molecule *i* to some cluster $C_{J}$ as follows:

(2) $$b_{i}=\min_{J\neq I}\frac{1}{|C_{J}|}\sum_{j\in C_{J}}d\left(i,j\right),$$

where d(i,j) is the distance between the molecule *i* to all molecules in $C_{J}$, with $C_{J}\neq C_{I}$.

Then, the silhouette coefficient is defined for each molecule using the mean intra-cluster distance $a_{i}$ and the mean inter-cluster distance $b_{i}$ as:

(3) $$SC\left(i\right)=\frac{b_{i}-a_{i}}{\max(a_{i},b_{i})}.$$

In summary, the silhouette coefficient is calculated using the mean distance between a given molecule and all other molecules in the same cluster $\left(a_{i}\right)$, and the mean distance between a given molecule and all other molecules in the next nearest-cluster $\left(b_{i}\right)$ [15]. The SC function implemented in the Scikit-learn library returns the mean silhouette coefficient over all molecules. The best value is 1, and the worst value is −1. Values near 0 indicate overlapping clusters. Negative values generally indicate that a molecule has been assigned to the wrong cluster as a different cluster is more similar.

The Calinski–Harabasz score [33], also known as the variance ratio criterion, is defined as the ratio of the sum of inter-clusters dispersion and of within-cluster dispersion for all clusters, where dispersion is defined as the sum of distances squared. This score is calculated for *k* clusters as follows:

(4) $$CH=\frac{tr(B_{k})}{tr(W_{k})}\times \frac{n_{E}-k}{k-1},$$

where the $tr(B_{k})$ is the trace of the between-cluster dispersion matrix, and $tr(W_{k})$ is the trace of the within-cluster dispersion matrix, defined by:

(5) $$W_{k}=\sum_{q=1}^{k}\sum_{x\in C_{q}}\left(x-c_{q}\right){\left(x-c_{q}\right)}^{T},$$

(6) $$B_{k}=\sum_{q=1}^{k}n_{q}\left(c_{q}-c_{E}\right){\left(c_{q}-c_{E}\right)}^{T},$$

with $C_{q}$ the set of points in cluster *q*, $c_{q}$ the center of cluster *q*, $c_{E}$ the center of cluster *E*, and $n_{q}$ the number of points in cluster *q*. A higher Calinski–Harabasz score relates to a model with better-defined clusters.

Finally, the Davies–Bouldin score [34] is defined as the average similarity measure of each cluster $C_{i}$ with its most similar cluster $C_{j}$:

(7) $$DB=\frac{1}{k}\sum_{i=1}^{k}\max_{i\neq j}R_{ij},$$

where $R_{ij}$ is defined as:

(8) $$R_{ij}=\frac{s_{i}+s_{j}}{d_{ij}},$$

and $s_{i}$ is the average distance between each molecule of cluster and the centroid of that cluster, and $d_{ij}$ is the distance between cluster centroids *i* and *j*. The minimum score is zero, with lower values indicating better clustering.

We complement this evaluation by calculating a matrix with the average Tanimoto similarity between the molecules of cluster *i* and those in cluster *j*. The average of these similarity scores represents the position (i,j) of the final matrix. In the case of average similarity between molecules from a cluster and itself (position (i,i)), the similarity was calculated between two different molecules. This means that values in position (i,i) do not include similarity scores of a molecule with itself. Note that the resulting matrix is a symmetric matrix (the similarity scores between cluster *i* and cluster *j* are equal to similarity scores between cluster *j* and cluster *i*); thus, for display purposes, the similarity matrix is shown as a lower triangular matrix.

## 3. Results

### 3.1. Outlier Detection

One of the aspects to consider when using ML-based algorithms, in particular those used for clustering, is that many of them are sensitive to outliers and fluctuations in the density of data points [35]. In this study, we defined an outlier as the molecule whose Tanimoto similarity value to their most similar molecule is lower than or equal to a given cutoff. Thus, an outlier molecule is different from any other molecule in the set at the predefined outlier cutoff.

To remove outlier molecules, we first calculated the Tanimoto similarity for each pair of molecules (1,277,878,735 pairs). Then, we retained the similarity value of each molecule to its closest molecule (other than itself). In this way, we only have 50,555 similarity values. We retain those molecules because all the other molecules will be even less similar. The next step was to find the best outlier cutoff based on the three clustering quality metrics. We used the hierarchical clustering (with *Ward linkage = 3*) as the baseline algorithm for this experiment and evaluated the outlier cutoffs from 0.2 to 0.5, with a step size of 0.1.

Table 1 shows that as we increase the outlier cutoff, we obtain fewer clusters. This may be because at each outlier cutoff we have fewer molecules to cluster, and they are more similar to each other. Additionally, increasing the outlier cutoff generally leads to all three metrics improving their results with respect to the previous outlier cutoff. Moreover, the outlier cutoff of 0.5 seems to be the best tradeoff between the number of clusters and their quality since the silhouette coefficient and the Davies–Bouldin score achieve better values at this outlier cutoff. Therefore, the outlier cutoff of 0.5 was used for the following experiments. Figure 3 shows the distribution of similarity values before and after the outlier detection method.

### 3.2. Hierarchical Clustering

To perform hierarchical clustering of molecules we have to specify the Ward linkage cutoff to be used. We evaluated the impact of different cutoffs on the three selected clustering quality metrics, as well as on the number of clusters obtained. The Ward linkage cutoffs explored ranged from 0.5 to 3.0, with a step size of 0.5. These cutoffs were applied to either all molecules or non-outlier molecules and are reported in Table 2. By using the Ward linkage as the merging criterion, we are requesting that at each step the hierarchical clustering algorithm has to find the pair of clusters that leads to the minimum increase in total intra-cluster variance after merging. Table 2 shows that stricter (smaller) Ward linkage cutoffs result in a larger number of clusters.

Regarding the clustering quality metrics, the silhouette coefficient suggests that some molecules have been assigned to the wrong cluster as negative values are obtained at each cutoff. The Calinski–Harabasz score suggests that better-defined clusters are obtained at higher cutoff values, 2.5 and 3.0 for instance. In contrast, the Davies–Bouldin score suggests that better-defined clusters are obtained at smaller cutoff values but at the cost of a larger number of clusters. This behavior is repeated for all molecules as well as for non-outlier molecules.

Considering that the main purpose of this clustering problem is to use the clusters for ML model validation, the results in Table 2 suggest that a cutoff value between 2.0 and 3.0 generates a number of clusters that is less computationally expensive to use in a cross-validation strategy. In particular, the Ward linkage cutoff of 3.0 achieves the best clustering quality results, and it improves between all molecules and non-outlier molecules. Indeed, at this cutoff the silhouette coefficient improves by 50%, the Calinski–Harabasz score improves by 10%, and the Davies–Bouldin score improves by 13% when using non-outlier molecules. Therefore, we chose the Ward linkage cutoff of 3.0 for the following experiments.

**Figure 4:**
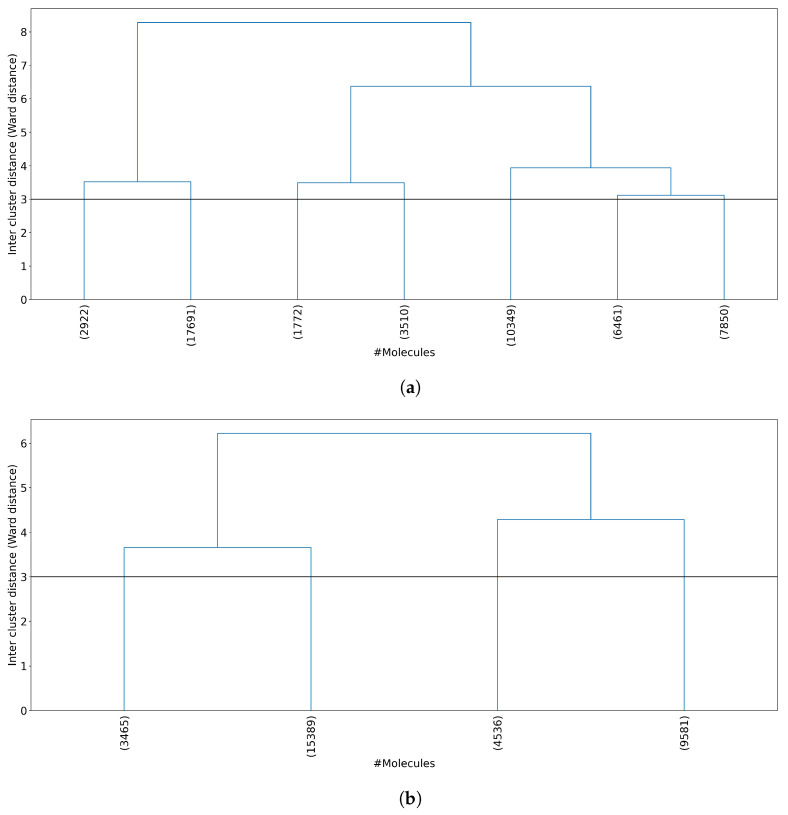
Dendrogram representation of hierarchical clustering results. (**a**) Clustering of all 50,555 molecules results in 7 clusters at a Ward linkage cutoff of 3. (**b**) Clustering of 32,971 non-outlier molecules results in 4 clusters at a Ward linkage cutoff of 3. For each plot, the horizontal axis shows the number of molecules per cluster, and the vertical axis shows the Ward linkage between any two clusters. Outlier molecules were removed using an outlier cutoff of 0.5 (see Section 3.1 for more details about outlier detection). The average cluster size is 7229 molecules (std = 5517) when all molecules are clustered and 8243 molecules (std = 5460) when non-outlier molecules are clustered.

The next step was to analyze the number of molecules per cluster. Figure 4 shows the dendrogram for both all 50,555 molecules and 32,971 non-outlier molecules. When all of the molecules are clustered (Figure 4a), one cluster concentrates about 35% of the molecules (17,691 molecules in cluster 2), whereas when non-outlier molecules are clustered (Figure 4b), one cluster concentrates about 46% of the molecules (15,389 molecules in cluster 2). Even with this high concentration of molecules in a single cluster, the remaining clusters have enough information as the smallest clusters obtained in all molecules and non-outlier molecules concentrate 3.5% and 10.5% of the molecules, respectively.

**Figure 5:**
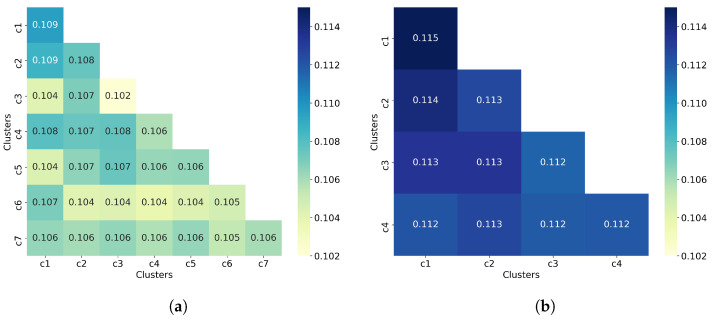
Hierarchical clustering with Ward linkage = 3. (**a**) Clustering of all 50,555 molecules results in 7 clusters. (**b**) The clustering of 32,971 non-outlier molecules results in 4 clusters. Outlier molecules were removed using an outlier cutoff of 0.5 (see Section 3.1 for more details about outlier detection). The matrix is calculated as the average Tanimoto similarity between the molecules of cluster *i* and those in cluster *j*, and the average similarity between molecules of a cluster and itself (see Section 2.3 for more details on how this matrix is calculated). The matrices have been adjusted to the same scale to facilitate clustering comparison between all molecules and non-outlier molecules. For readability, similarity scores were rounded to three decimal places. The average similarity between a cluster and itself (diagonal) is close to the average similarity between two different clusters (off-diagonal), indicating poor clustering of both all molecules and non-outlier molecules.

To complement the analysis of the clustering quality, we now calculate the average similarity matrix for both all molecules and non-outlier molecules. This matrix was calculated as explained in Section 2.3. In a good clustering result, molecules from different clusters should have much lower similarity than molecules from the same cluster. In the average similarity matrix, this means that the similarity values on the diagonal must always be greater than the off-diagonal values. Figure 5 shows that molecules from the same cluster are, on average, very similar to molecules from different clusters since the off-diagonal values are mostly equal to the diagonal values, and in some cases even greater than the diagonal values. This suggests a poor clustering of molecules by using hierarchical clustering (with Ward linkage = 3), even when outlier molecules are removed. This assumption is consistent with the values of the clustering quality metrics reported in Table 2.

### 3.3. Taylor–Butina Clustering

One of the advantages of Taylor–Butina clustering is that molecules in the resulting clusters will have a Tanimoto value greater than or equal to the established similarity cutoff. In this study, we analyzed similarity cutoffs 0.35, 0.5, 0.6, 0.7, 0.8, 0.9, 0.95, 0.97, and 0.99. These cutoffs were applied to either all molecules or non-outlier molecules and evaluated in terms of the three clustering quality metrics.

Table 3 shows that smaller similarity cutoffs result in a larger number of clusters. As for the clustering quality metrics, the silhouette coefficient and the Davies–Bouldin score suggest that better-defined clusters are obtained with smaller similarity cutoff values but at the cost of a higher number of clusters. This trend is observed for all molecules as well as for non-outlier molecules. The number of clusters should also be considered as it impacts the number of cross-validations and hence the computational cost. Therefore, if we consider the number of clusters then the Calinski–Harabasz score suggests that the similarity cutoff of 0.9 is the best option. Overall, the three clustering quality metrics improve between all molecules and non-outlier molecules.

Since one of the drawbacks of the Taylor–Butina algorithm is the possibility of obtaining clusters that are singletons, the next step in this analysis is to evaluate the number of molecules per cluster. Based on results from Table 3, we choose the similarity cutoff of 0.9 to perform this experiment. Indeed, Table 4 shows that when all molecules are clustered, 17% of the clusters obtained are singletons. This value decreases to 10% in the non-outlier molecules. Moreover, 90% of all molecules and 93% of the non-outlier molecules have been assigned to a single cluster.

**Figure 6:**
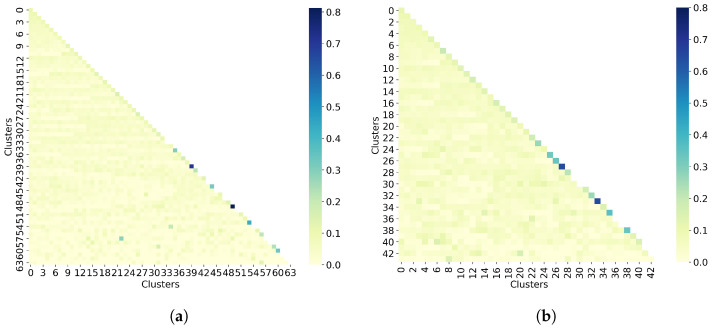
Taylor-Butina clustering with similarity cutoff = 0.90. (**a**) Clustering of all 50,555 molecules results in 64 clusters, and (**b**) clustering of 32,971 non-outlier molecules results in 44 clusters. Outlier molecules were removed using an outlier cutoff of 0.5 (see Section 3.1 for more details about outlier detection). The matrix is calculated as the average Tanimoto similarity between the molecules of cluster *i* and those in cluster *j*, and the average similarity between molecules of a cluster and itself (see Section 2.3 for more details on how this matrix is calculated). The matrices have been adjusted to the same scale to facilitate clustering comparison between all molecules and non-outlier molecules. The average similarity between one cluster and itself (diagonal) is substantially higher than the average similarity between two different clusters (off-diagonal). However, these values may be biased by the number of molecules per cluster.

The last step in the clustering quality analysis is to calculate the average similarity matrix for both all molecules and non-outlier molecules. To analyze these matrices, which are calculated as the average within-cluster and between-cluster similarity, the sizes of the clusters must be taken into account. Indeed, since both cases (all molecules and non-outlier molecules) have singletons, it is not possible to calculate the average within-cluster similarity, so these values are missing on the matrix diagonal. For the average between-cluster similarity, it can be the similarity between two molecules. Overall, Figure 6 shows that molecules from different clusters have lower similarity than molecules from the same cluster, notably for non-outlier molecules. However, these values may be biased by the number of molecules per cluster.

### 3.4. UMAP Clustering

UMAP has four basic hyperparameters (n_neighbors, min_dist, n_components, and metric) that control the dimensionality reduction result and one that controls the clustering (n_clusters). In this study, MFPs are embedded in two dimensions (n_components=2) and the Jaccard metric is used to calculate the distance between MFPs. For the remaining hyperparameters (n_neighbors, min_dist, and n_clusters), we implemented a two-step hyperparameter tuning process to find their optimal values. In the first step, we fixed the number of clusters and tune the values of the number of neighbors (n_neighbors) and the distance (min_dist). Based on the results obtained with hierarchical clustering, when all molecules are clustered, the number of clusters was set to 7. The evaluation of this experiment was done in terms of the three clustering quality metrics. Once these hyperparameters have been set, the second step of hyperparameter tuning uses these values and focuses on finding the optimal number of clusters by using the *elbow* method [36]. The elbow method fits the clustering model for a range of n_clusters values. If the data are very clustered, the optimal number of clusters is given by the point of inflection on the curve (i.e., the elbow); otherwise, the elbow will be unclear.

**Figure 7:**
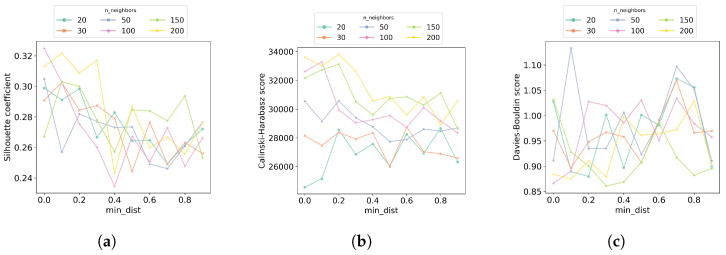
Increasing the number of neighbors leads to recovering better-defined clusters. In the first step of the hyperparameter tuning process for UMAP clustering, a grid search was performed to find the best combination of the number of neighbors (n_neighbors) and distance (min_dist). The number of clusters used for this search was set to 7. Each combination of (min_dist, n_neighbors) values was evaluated considering the clustering quality metrics (**a**) silhouette coefficient, (**b**) Calinski–Harabasz score, and (**c**) Davies–Bouldin score. For each plot, the horizontal axis shows the distance values (min_dist) evaluated, the vertical axis shows the corresponding metric values, and the color code identifies the number of neighbors (n_neighbors) evaluated.

In the first step of the hyperparameter tuning process, we performed a grid search to look for the best n_neighbors and min_dist hyperparameters. The values of n_neighbors evaluated are 20, 30, 50, 100, 150, and 200, while for min_dist the values evaluated are in the range of 0.0 to 0.9, with a step size of 0.1. Figure 7 shows the results of each of the clustering quality metrics obtained at each hyperparameter combination. Overall, the results suggest that using 100 or 200 neighbors UMAP is able to balance local versus global structure in the molecules in a better way than with fewer neighbors. Additionally, at these numbers of neighbors, the best min_dist is 0.0, which means molecules represented in the embedded space are close to each other. Since the combination (100, 0.0) is the best in two of the three clustering quality metrics, these values were used in the second step of the hyperparameter tuning.

Given the values n_neighbors=100 and min_dist=0.0, we now look for the optimal number of clusters to be used. Since our evaluation of clustering quality goes beyond the three clustering metrics, we use the elbow method to narrow the search for the number of clusters rather than to find the optimal number. Here, we explored the range of clusters from 2 to 25. We also compared two scoring metrics to evaluate the clusters. These scoring metrics are the distortion, defined by the sum of squared distances between each observation and its closest centroid, and the silhouette metric, defined by the mean ratio of intra-cluster and nearest-cluster distance.

**Figure 8:**
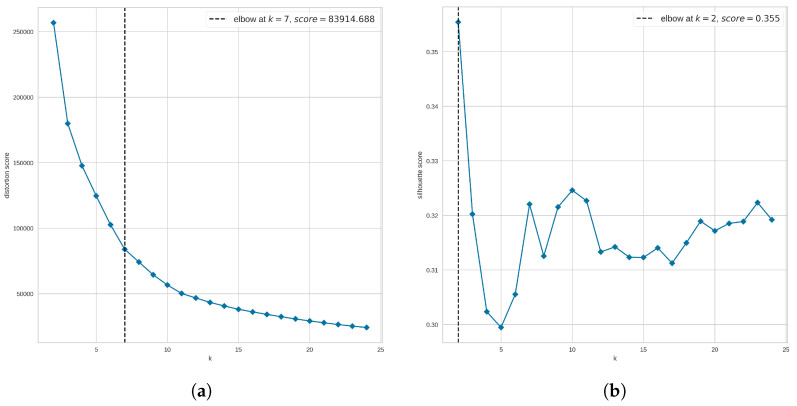
Elbow method to narrow the search for the optimal number of clusters when using UMAP clustering. In the second step of the hyperparameter tuning process for UMAP clustering, the elbow method was explored, covering a range of clusters from 2 to 25, and using (**a**) the distortion metric and (**b**) the silhouette metric. For each plot, the horizontal axis shows the number of clusters (*k*) evaluated, and the vertical axis shows the corresponding metric.

Figure 8a suggests that the optimal number of clusters is 7 when using the distortion score. However, Figure 8b suggests that our data are not highly clustered as the elbow is not clearly defined when the Silhouette score is used. Instead, the method returns as the optimal number of clusters the value at which the highest Silhouette score was obtained; in this case, the suggested optimal number is 2 clusters. Since we are using the elbow method to narrow the search for the optimal number of clusters, from Figure 8 we analyze clusters 6, 7, and 8. In addition, to find out whether a larger number of clusters improves the clustering results, we also explored the UMAP clustering when 20 groups are requested. These values were applied to either all molecules or non-outlier molecules and evaluated in terms of the three clustering quality metrics.

With the best combination of hyperparameters for UMAP found, we evaluated the clustering results in terms of the clustering quality metrics obtained at each number of clusters. Table 5 shows these results, where, in summary, all clustering quality metrics suggest well-defined clusters. Even when all metrics reach their best value with different numbers of clusters, the difference in the results with respect to the best value is small. In fact, the difference of the best silhouette coefficient with respect to the other values is between 3–6%; for the Calinski–Harabasz score, this difference ranges from 0.5% to 12%; and for the Davies–Bouldin score the difference ranges from 2% to 4%. For non-outlier molecules, the difference between the best and worst value for each of the clustering metrics is between 4.5 and 11% for the silhouette coefficient, between 6 and 12% for the Calinski–Harabasz score, and between 1 and 5.5% for the Davies–Bouldin score.

The next step was to analyze the number of molecules per cluster when 7 and 20 clusters are requested. These two values were selected considering the optimal number of clusters obtained with the elbow method (Figure 8a) and the results of the clustering metrics (Table 5). Table 6 shows the descriptive statistics of the cluster sizes, for both all 50,555 molecules and 32,971 non-outlier molecules. In general, the size of the clusters is well distributed (mean cluster size is close to median cluster size) for all molecules as well as for non-outlier molecules. Moreover, each of the 7 clusters concentrates between 5 and 22% of all molecules, and between 4 and 24% of non-outlier molecules. However, when 20 clusters are required, the smallest clusters in each case concentrate 1.19% and 0.96% of the molecules, respectively. This should be taken into account when performing cross-validation since having few molecules could affect the model performance.

**Figure 9:**
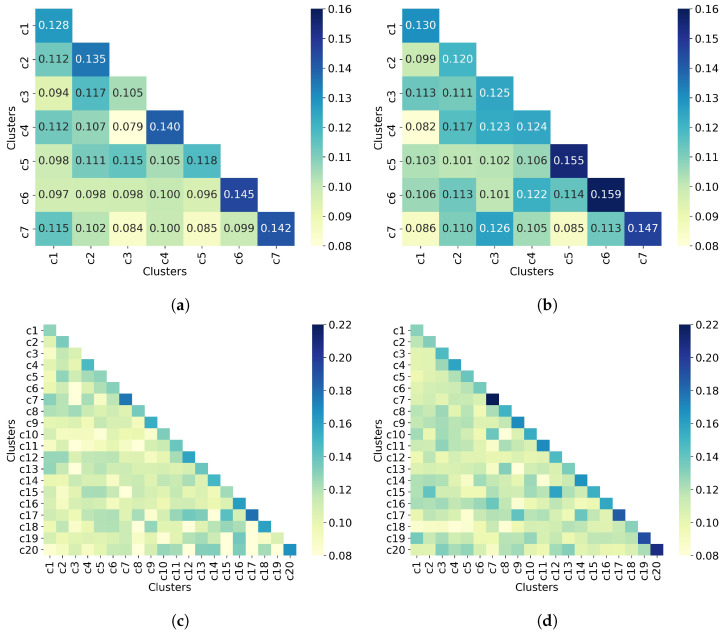
UMAP clustering with *n_neighbors* = 100 and *min_dist* = 0.0. Clustering of (**a**) all 50,555 molecules into 7 clusters, (**b**) 32,971 non-outlier molecules into 7 clusters, (**c**) all 50,555 molecules into 20 clusters, and (**d**) 32,971 non-outlier molecules into 20 clusters. Outlier molecules were removed using an outlier cutoff of 0.5 (see Section 3.1 for more details about outlier detection). The matrix is calculated as the average Tanimoto similarity between the molecules of cluster *i* and those in cluster *j*, and the average similarity between molecules of a cluster and itself (see Section 2.3 for more details on how this matrix is calculated). The matrices have been adjusted to the same scale to facilitate clustering comparison between all molecules and non-outlier molecules. For readability, similarity scores were rounded to three decimal places when seven clusters were requested. When 20 clusters were requested, the similarity scores are not displayed. The average similarity between one cluster and itself (diagonal) is substantially higher than the average similarity in different clusters (off-diagonal), indicating well-defined clusters in both all molecules and non-outlier molecules.

The last step in the clustering quality analysis is to calculate the average similarity matrix for both all molecules and non-outlier molecules. Overall, Figure 9 shows that molecules from different clusters have lower similarity than molecules from the same cluster, which indicates good clustering results. This is consistent with the clustering quality metrics shown in Table 5. Moreover, the similarity values between a cluster and itself (diagonal) improve when only non-outlier molecules are clustered. As for the number of clusters, increasing this number (from 7 to 20) increases the similarity between a cluster and itself from 0.16 to 0.22, for the upper bound.

### 3.5. Comparison of Clustering Algorithms

Finally, we compare the best clustering results obtained with each of the three methods explored in this study. In the case of UMAP clustering, we compare the results when 7 and 20 clusters are required. This comparison is performed for all molecules as well as for non-outlier molecules. Additionally, we include the run time (in minutes) taken by each method. Table 7 summarizes the results of this comparison.

When all molecules are clustered, the UMAP model obtains the best results on all metrics, whether using 7 or 20 clusters. Indeed, the silhouette coefficient improves between 0.339 and 0.521 units with respect to hierarchical and Taylor–Butina clustering. With respect to the Calinski–Harabaz score, although there is no upper limit for this score, the improvement is evident, with more than 34K units. As for the Davies–Bouldin score, the improvement is between 3–76 units. Regarding the run time, the Taylor–Butina clustering consumes 10x more time than the UMAP clustering, which is the fastest method. Similar behavior occurs when non-outlier molecules are clustered. That is, the UMAP clustering achieves the best results in terms of the three quality metrics as well as the run time.

In general, removing outlier molecules prior to clustering leads to an improvement in hierarchical and UMAP clustering (with 20 clusters). In the case of the Taylor–Butina clustering, there is no improvement by performing this step; in fact, the silhouette coefficient decreases by 2.5%, the Calinski–Harabasz score decreases by 17%, and the Davies–Bouldin score decreases by 1.3%. The absence of improvement in UMAP with seven clusters when outliers are removed may be due to the fact that according to the elbow method (Figure 8a), this is the optimal number of clusters, and therefore an improvement could be difficult to achieve.

## 4. Discussion

The main aim of this study is to provide an optimal clustering of molecules from the NCI-60 panel, which can be used to generate clusters for model validation. As model validation is a technique to assess model generalization, high-quality clustering results could improve the generalization of ML-based models. However, in studies using clustering methods that derive clusters to be used in cross-validation (LOCCO-CV, for example), the analysis of clustering quality is usually omitted and at best restricted to a single metric. To provide a comprehensive comparison of the clustering results obtained by hierarchical, Taylor–Butina, and UMAP clustering, in this study we show three well-known clustering metrics, along with the similarity matrix. The latter provides information on the structure of each cluster obtained.

The results show that the clustering quality metrics vary from method to method (Table 2, Table 3 and Table 5). Using different cutoffs in hierarchical and Taylor–Butina clustering leads to results that are computationally expensive to use under a cross-validation strategy. Even at cutoff values where there is a trade-off between the number of clusters obtained and the quality of the clustering, according to the similarity matrix (Figure 5 and Figure 6), the molecules in different clusters are similar to each other. This inter-cluster similarity may affect the model performance since similar molecules are present in the training set and the test set. This highlights the importance of complementing the usual clustering metrics by calculating the average similarity matrix.

In methods such as hierarchical or Taylor–Butina clustering, the number of clusters is not an input but a consequence of the selected cutoff level. In the case of UMAP clustering, the number of clusters must be provided by the user. Since this parameter can affect the clustering quality, we address this problem by using the elbow method. However, instead of using it to find the optimal number of clusters, we use it to narrow the search for this number. The results suggest that we can generally get higher-quality clustering if we request a higher number of clusters. However, we require a compromise between the number of clusters and clustering quality as more clusters imply more cross-validations and thus more expensive computation.

In addition to the optimal number of clusters, the elbow method also provides insights into the clusterability of the data set (Figure 8). In this case, this analysis suggests that the molecules in the NCI-60 panel are not very clustered as the inflection point of the curve is not clearly observed (Figure 8b). This has a greater impact on hierarchical and Taylor–Butina clustering, in addition to the similarity distribution of molecules (Figure 3), since these methods also require a cutoff that determines the behavior of the clusters (intra-cluster distance or similarity between molecules).

Hierarchical, Taylor–Butina, and UMAP clustering were also tested to evaluate if the removal of outlier molecules improves clustering quality. We define outlier molecules and evaluate the results according to the desired clustering. In this case, the results demonstrate a benefit when non-outlier molecules are clustered using hierarchical and UMAP clustering. This is not the case for the Taylor–Butina clustering, where a decrease in the metrics is observed (Table 7). This suggests that Taylor–Butina is not suitable for clustering molecules having a distribution as shown in Figure 3 since in both scenarios (all molecules or non-outlier molecules) we obtain clusters that concentrate most of the molecules or singletons. While the adopted outlier detection method improves clustering, we believe that a comprehensive search for an optimal method using high-quality packages such as PyOD [37] is likely to result in further improvement.

In summary, there are many factors influencing clustering problems, particularly the clustering of molecules. Models that are not properly validated are susceptible to reduced performance in unseen compounds; therefore, poor clustering of molecules can lead to the poor estimation of model generalizability. Since results showed different clustering quality metrics with respect to the method used, special care must be taken in this task.

## 5. Conclusions

- In this paper, we provide a comparison of hierarchical, Taylor–Butina, and UMAP clustering to find the best method to cluster NCI-60 molecules. We also evaluated the impact of removing outlier molecules before clustering for each of these methods.
- Results suggest that the most effective way to cluster the NCI-60 molecules is by using the UMAP algorithm either with 7 or 20 clusters depending on the clustering quality metric selected. In addition, the choice of the number of clusters to be used must be balanced with the computational cost that the user can afford since more clusters imply more cross-validations.
- All three clustering methods have been tested on other datasets; however, we are not aware of any previous studies that have clustered all or part of the molecules from the NCI-60 panel nor of studies comparing results when outlier molecules are removed.
- High-quality clustering results could improve model generalization on unseen compounds, which is important when the scope and use of the predictive model need to be extended. Therefore, special care must be taken in the clusters to be used in model validation, such as LOCCO-CV strategies.
- In this study, the 1024-bit Morgan fingerprint was utilized as a descriptor to characterize the molecules in the NCI-60 panel. Although this is likely to be a near-optimal choice given past comparative studies, future work could investigate other descriptors to find out whether they improve the clustering of the NCI-60 molecules further.
- Another interesting topic for future work is to investigate how clustering is affected by the inclusion of large proportions of assumed inactive molecules (decoys) generated for each NCI-60 molecule with potent activity as it wis important that decoys are contained in the same cluster as the active they were generated from [38].

## Figures and Tables

**Table 1:** Hierarchical clustering (with Ward linkage = 3) to identify the best outlier cutoff. Increasing the outlier cutoff, and thus removing more molecules, leads to better quality clustering according to the three selected metrics. Comparison of outlier cutoffs based on the number of molecules removed and the clustering metrics. The best value for each metric is highlighted in bold. For readability, clustering quality results were rounded to either two or three decimal places.

Outlier Cutoff	Molecules Removed	# Clusters	Silhouette Coefficient	Calinski–Harabasz Score	Davies–Bouldin Score
0.2	151	6	−0.011	5.10	87.72
0.3	2096	6	−0.013	4.65	79.91
0.4	8994	5	−0.006	**5.45**	84.81
0.5	17,584	4	**−0.007**	5.32	**67.15**

**Table 2:** Clustering quality depending on cutoff and outlier removal. Overall, an improvement in each of the clustering quality metrics is observed when outlier molecules are removed. Clustering quality metrics were calculated for each Ward linkage cutoff evaluated in hierarchical clustering. From top to bottom, each cutoff is stricter than the previous one. Here, 32,971 non-outlier molecules were retrieved at an outlier cutoff of 0.5. The best value for each metric for either all molecules or non-outlier molecules is highlighted in bold. For readability, clustering quality results were rounded to either two or three decimal places.

	All Molecules				Non-Outlier Molecules			
**Ward Linkage Cutoff**	**# Clusters**	**Silhouette Coefficient**	**Calinski–Harabasz Score**	**Davies–Bouldin Score**	**# Clusters**	**Silhouette Coefficient**	**Calinski–Harabasz Score**	**Davies–Bouldin Score**
3.0	7	**−0.014**	**4.84**	77.19	4	**−0.007**	**5.32**	67.15
2.5	9	**−0.014**	4.01	80.19	7	−0.009	3.50	69.94
2.0	89	−0.022	1.77	28.63	88	−0.030	1.96	21.45
1.5	2565	−0.106	1.28	7.28	1766	−0.103	1.37	6.97
1.0	17,958	−0.257	1.05	2.59	11,436	−0.260	1.07	2.62
0.5	33,246	−0.305	1.01	**1.43**	20,723	−0.339	1.01	**1.53**

**Table 3:** Better-defined clusters are obtained when using smaller similarity cutoff values, at the cost of a larger number of clusters. Clustering quality metrics were calculated for each similarity cutoff evaluated in the Taylor–Butina clustering. Non-outlier molecules were retrieved at an outlier cutoff of 0.5. The best value for each metric for either all molecules or non-outlier molecules is highlighted in bold. For readability, clustering quality results were rounded to either two or three decimal places.

	All Molecules				Non-Outlier Molecules			
**Similarity Cutoff**	**# Clusters**	**Silhouette Coefficient**	**Calinski–Harabasz Score**	**Davies–Bouldin Score**	**# Clusters**	**Silhouette Coefficient**	**Calinski–Harabasz Score**	**Davies–Bouldin Score**
0.35	30,590	**0.111**	6.10	**0.65**	16,860	**0.120**	7.09	**0.72**
0.50	19,513	0.086	5.27	0.93	9077	0.092	7.06	1.06
0.60	12,797	0.029	4.95	1.20	5996	0.042	6.83	1.30
0.70	5872	−0.067	4.96	1.66	2984	−0.046	6.54	1.74
0.80	1074	−0.168	6.64	2.61	634	−0.155	7.38	2.63
0.90	64	−0.196	**9.03**	3.78	44	−0.201	**7.48**	3.83
0.95	21	−0.204	2.85	3.27	11	−0.203	2.25	2.73
0.97	12	−0.214	1.40	2.51	5	−0.202	0.76	1.96
0.99	4	−0.215	0.65	2.12	1	–	–	–

**Table 4:** Descriptive statistics of the cluster sizes obtained by the Taylor–Butina algorithm. This algorithm was applied to all molecules and non-outlier molecules, showing that singletons were obtained in both cases. The similarity cutoff used to obtain the clusters in Taylor–Butina was set to 0.9 (see Table 3 for more details about the similarity cutoff selected). Non-outlier molecules were retrieved at an outlier cutoff of 0.5.

	All Molecules	Non-Outlier Molecules
Number of clusters	64	44
mean	790	749
std	5703	4617
min	1	1
10%	1	1
17%	1	2
20%	2	2
25%	3	3
50%	7	13
75%	52	30
max	45,652	30,669

**Table 5:** Clustering quality metrics suggest well-defined clusters at each selected number of clusters. Clustering quality metrics were calculated for each number of clusters evaluated in the UMAP algorithm. Non-outlier molecules were retrieved at an outlier cutoff of 0.5. The best value for each metric for either all molecules or non-outlier molecules is highlighted in bold. For readability, clustering quality results were rounded to either two or three decimal places.

	All Molecules			Non-Outlier Molecules		
**Number of Clusters**	**Silhouette Coefficient**	**Calinski Harabasz Score**	**Davies Bouldin Score**	**Silhouette Coefficient**	**Calinski Harabasz Score**	**Davies Bouldin Score**
6	0.306	30,410.88	0.90	0.318	20,683.06	**0.85**
7	**0.325**	32,621.97	0.87	0.295	21,420.86	0.89
8	0.314	34,263.14	**0.86**	0.302	22,194.48	0.90
20	0.315	**34,464.21**	0.88	**0.333**	**23,609.54**	0.86

**Table 6:** Descriptive statistics of the cluster sizes obtained by the UMAP clustering. This algorithm was applied to all molecules and non-outlier molecules show that the size of the clusters is well distributed in both cases. Non-outlier molecules were retrieved at an outlier cutoff of 0.5. The number of clusters required was set to either 7 or 20 (see Table 5 for more details about the number of clusters selected).

	All Molecules		Non-Outlier Molecules	
# of clusters	7	20	7	20
mean	7222	2528	4710	1649
std	3575	1032	2149	846
min	2664	605	1811	318
25%	4444	1981	3241	884
50%	6960	2486	4684	1524
75%	10,522	3202	5961	2391
max	11,000	4364	8072	3058

**Table 7:** UMAP clustering outperforms results obtained by hierarchical and Taylor–Butina clustering. Comparison of the clustering quality metrics for each clustering algorithm. Non-outlier molecules were retrieved at an outlier cutoff of 0.5. The best value for each metric for either all molecules or non-outlier molecules is highlighted in bold. For readability, clustering quality results were rounded to either two or three decimal places.

	All Molecules					Non-Outlier Molecules				
**Clustering Method**	**Number of Clusters**	**Silhouette Coefficient**	**Calinski Harabasz Score**	**Davies Bouldin Score**	**Time Took**	**Number of Clusters**	**Silhouette Coefficient**	**Calinski Harabasz Score**	**Davies Bouldin Score**	**Time Took (min)**
HC	7	−0.014	4.84	77.19	25	4	−0.007	5.32	67.15	3
TB	64	−0.196	9.03	3.78	40	44	−0.201	7.48	3.83	6
UMAP	7	**0.325**	32,621.97	**0.87**	11	7	0.295	21,420.86	0.89	2
UMAP	20	0.315	**34,464.21**	0.88	4	20	**0.333**	**23,609.54**	**0.86**	2

## Data Availability

The code for the reproduction of the best clustering results is available at https://github.com/Sahet11/nci60_clustering, accessed on 1 February 2023.

## Abbreviations

The following abbreviations are used in this manuscript:

AI	Artificial intelligence
ML	Machine learning
QSAR	Quantitative structure–activity relationships
AVE	Asymmetric validation embedding
HTS	High-throughput screening
CV	Cross-validation
LDTO	Leave-dissimilar-target-out
LOTO	Leave-one-tissue-out
LOCCO	Leave-one-compound-cluster-out
LOCO	Leave-one-cell-line-out
MOA	Mechanism of action
NCI	National Cancer Institute
NSC	National Service Center
MFP	Morgan fingerprint
SMILES	Simplified molecular-input line-entry system
UMAP	Uniform manifold approximation and projection
HC	Hierarchical clustering
TB	Taylor–Butina
SC	Silhouette coefficient
CH	Calinski–Harabasz score
DB	Davies–Bouldin score
